# Supersaturation and Solubilization upon In Vitro Digestion of Fenofibrate Type I Lipid Formulations: Effect of Droplet Size, Surfactant Concentration and Lipid Type

**DOI:** 10.3390/pharmaceutics13081287

**Published:** 2021-08-18

**Authors:** Vladimir Katev, Sonya Tsibranska-Gyoreva, Zahari Vinarov, Slavka Tcholakova

**Affiliations:** Department of Chemical and Pharmaceutical Engineering, Faculty of Chemistry and Pharmacy, Sofia University, 1164 Sofia, Bulgaria; vk@lcpe.uni-sofia.bg (V.K.); st@lcpe.uni-sofia.bg (S.T.-G.); sc@lcpe.uni-sofia.bg (S.T.)

**Keywords:** oral absorption, emulsion, precipitation, lipolysis

## Abstract

Lipid-based formulations (LBF) enhance oral drug absorption by promoting drug solubilization and supersaturation. The aim of the study was to determine the effect of the lipid carrier type, drop size and surfactant concentration on the rate of fenofibrate release in a bicarbonate-based in vitro digestion model. The effect of the lipid carrier was studied by preparing type I LBF with drop size ≈ 2 µm, based on medium-chain triglycerides (MCT), sunflower oil (SFO), coconut oil (CNO) and cocoa butter (CB). The drop size and surfactant concentration effects were assessed by studying MCT and SFO-based formulations with a drop size between 400 nm and 14 µm and surfactant concentrations of 1 or 10%. A filtration through a 200 nm filter followed by HPLC analysis was used to determine the aqueous fenofibrate, whereas lipid digestion was followed by gas chromatography. Shorter-chain triglycerides were key in promoting a faster drug release. The fenofibrate release from long-chain triglyceride formulations (SFO, CNO and CB) was governed by solubilization and was enhanced at a smaller droplet size and higher surfactant concentration. In contrast, supersaturation was observed after the digestion of MCT emulsions. In this case, a smaller drop size and higher surfactant had negative effects: lower peak fenofibrate concentrations and a faster onset of precipitation were observed. The study provides new mechanistic insights on drug solubilization and supersaturation after LBF digestion, and may support the development of new in silico prediction models.

## 1. Introduction

The prevalence of poorly water-soluble drugs (PWSD) among new drug candidates that have emerged from modern drug discovery pipelines has put a significant pressure on oral drug development [1,2,3] due to the poor or highly variable absorption of these compounds [4,5,6]. One of the drug delivery technologies that manages to overcome the poor biopharmaceutical properties of PWSD are the lipid-based formulations (LBF) [7,8].

LBF address the oral delivery of PWSD by dissolving the drug in a mixture of glycerides, surfactants and/or co-solvents, hence introducing it in the gastrointestinal tract as a lipid solution [9,10,11]. A rough relationship between LBF composition and their dispersion and digestion behavior is provided by the lipid formulation classification system [12]. According to this classification, type I formulations are composed by glycerides, disperse poorly in intestinal fluids and the drug release depends on the lipolysis of the carrier. The addition of surfactants (type II, III and IV LBF) and co-solvents (type III and IV LBF) results in self-emulsifying formulations, which have better dispersion properties and rely less on digestion for drug release. However, the increased dispersibility usually comes at the cost of a decreased solubilization capacity, which can drive undesired drug precipitation [12].

LBF increase the oral bioavailability of PWSD by several complementary mechanisms [7,13,14], one of which is by improving drug solubilization in the small intestinal fluids [15,16,17,18,19]. The impact of lipid digestion products on the drug solubilization capacity of bile salt–phospholipid mixtures has been confirmed by many in vitro studies [20,21,22,23,24,25,26,27], leading to the introduction of sodium oleate and glycerol monooleate as components of fed state simulated intestinal fluids [28]. Somewhat surprisingly, despite the large number of in vitro lipolysis studies of drug release from LBF [29,30,31,32,33,34,35,36,37,38,39,40,41,42], so far only two studies have attempted to correlate the concentration of the solubilized drug with the concentrations of lipid digestion products in the aqueous phase [29,30].

Zangenberg et al. determined the total fatty acids (FA), monoglycerides (MG) and diglycerides (DG) obtained after the in vitro lipolysis of drug-loaded soybean oil emulsions by thin layer chromatography [30]. The authors studied the correlation between the aqueous drug and the sum of bile salt, FA and MG concentrations in the aqueous phase and found an excellent (*R*^2^ = 0.99) correlation for danazol, whereas worse results were observed for probucol (*R*^2^ = 0.79). The behavior of the two drugs was explained with differences in the forces that drive the transfer to the aqueous phase: a high affinity for the mixed bile salt–lipid micelles for danazol, compared to the reduced oil volume forcing drug release for probucol [30].

In a more recent study, Dening et al. determined the concentration and phase distribution of lipids obtained after the in vitro digestion of medium-chain triglyceride (MCT)-loaded inorganic particles by ^1^H NMR spectroscopy [29]. Although the considered sample set was small (*n* = 4), an excellent correlation (*R*^2^ = 0.996) between coumarin 102 solubilization and FA concentration in the aqueous phase was observed.

Apart from solubilization, the increased intestinal absorption of LBF has also been linked to the formation of transiently stable supersaturated drug solutions upon the digestion or dispersion of LBF [43,44], and has prompted research on pre-supersaturated formulations [45,46,47,48,49]. The metastable nature of supersaturated solutions [50,51,52,53,54] suggests that the rate of mass transfer of drug molecules from the LBF to the various phases (aqueous, solid or micellar) could significantly affect the supersaturation time window, as well as the drug phase distribution. One of the factors that is known to impact drug release from lipid vehicles is the specific surface area [55], which is determined by the drop size of the emulsion that is formed after dispersion in the gastrointestinal fluids.

Although the in vitro methodology for LBF characterization has evolved [40,56,57,58] and many aspects of LBF performance have been explored [39,41,42], clear conclusions about the impact of the drop size on drug solubilization and supersaturation cannot be drawn based on existing data. This is due to the fact that the drop size of LBF is varied by altering the excipient types and concentrations (or by switching from type I to type IV LBF) [59,60,61,62], which also brings changes to the solvent capacity of the LBF and its solubilization capacity after dispersion/digestion. Hence, the effect of LBF drop size is usually convoluted with the effects of LBF composition. This inherent ambiguity in the interpretation of drop size effects may explain why a smaller drop size does not always lead to increased bioavailability (as demonstrated for seocalcitol and danazol [60,63]), despite the perceived superior performance of self-nano-emulsifying and self-microemulsifying drug delivery systems (SNEDDS and SMEDDS) of cinnarizine, cyclosporine and penclomedine [64,65,66,67].

Therefore, we aimed to determine the effect of the drop size on drug release and supersaturation for type I LBF. As the surfactant concentration is another important variable in LBF development, its impact was assessed as well. Fenofibrate was chosen as a model drug due to its lipophilic nature, its frequent use in LBF studies [18,19,31,41,42,59,62] and the recently published data on the impact of polar lipids on its solubilization in biorelevant media [22]. Fenofibrate solutions were prepared in pure oils and fats, which were then emulsified at different emulsifier concentrations. Three emulsification methods were used in order to obtain initial drop diameters covering both crude emulsions and nanoemulsions, which were introduced in an in vitro digestion model. The lipid digestion products generated during in vitro lipolysis were determined via gas chromatography (GC), while fenofibrate was quantified by HPLC. This allowed us to correlate drug solubilization with the concentration of lipolysis products. Supersaturation was assessed by comparing the measured aqueous fenofibrate to the solubilization capacity of the digests.

## 2. Materials and Methods

### 2.1. Materials

Fenofibrate (99%) was obtained from Sigma-Aldrich (Saint Louis, MO, USA). Lipid formulations of fenofibrate were prepared in four lipid carriers: sunflower oil (SFO, Billa, Sofia, Bulgaria), medium-chain triglycerides (Kollisolv^®^ MCT 70, kindly donated by BASF, Ludwigshafen, Germany), coconut oil (CNO, kindly donated by Unilever, Colworth, UK) and cocoa butter (CB, obtained from Chemax pharma, Sofia, Bulgaria). The FA profile of the used fats and oils was determined in-house by GC and was used to calculate the average triglycerides (TG) molecular weight (see Table 1). Polysorbate 20 (Sigma-Aldrich, Saint Louis, MO, USA) was used as emulsifier for the preparation of oil-in-water emulsions. Sodium benzoate (99%, Sigma-Aldrich, Saint Louis, MO, USA) was used as a preservative in the emulsions. Sodium chloride (99%, Sigma-Aldrich, Saint Louis, MO, USA), potassium chloride (99%, Valerus, Sofia, Bulgaria), calcium chloride (99%, Merck, Darmstadt, Germany), sodium bicarbonate (99%, Teokom, Sofia, Bulgaria) and hydrochloric acid (37%, Sigma-Aldrich, Saint Louis, MO, USA) were used as electrolytes and pH control agents in the in vitro digestion model. Pepsin from porcine gastric mucosa, pancreatin from porcine pancreas (4 × USP specifications) and porcine bile extract (all products of Sigma-Aldrich, Saint Louis, MO, USA) were used to mimic enzymatic hydrolysis and solubilization during gastrointestinal digestion. The porcine bile extract contained 50 wt% bile acids (average molecular mass of 421 g/mol [68]), 6 wt% phosphatidylcholine and less than 0.06 wt% Ca^2+^ [69]. In-house analysis by GC showed that the used batch of porcine bile (MKBQ8333V) also containe 3.45 wt% FA and 0.77 wt% cholesterol. N,O-Bis(trimethylsilyl)trifluoroacetamide (BSTFA, reagent grade, Sigma-Aldrich, Saint Louis, MO, USA) was used for derivatization of the samples analyzed by GC. Methanol, chloroform, pyridine and isooctane (all products with purity >99% of Sigma-Aldrich, Saint Louis, MO, USA) were used as solvents for chromatographic analysis.

### 2.2. Emulsion Preparation and Characterization

#### 2.2.1. Aqueous and Oily Phase Preparation

The aqueous and oily phases used for preparation of the fenofibrate-loaded oil-in-water emulsions were prepared as follows. For the emulsions prepared by sonication, the polysorbate 20 emulsifier was dissolved in water at a concentration of 1 wt%. In order to prepare nanoemulsions, we had to increase the emulsifier concentration in the aqueous phase to 10 wt% in order to cover the large surface area created during high-pressure homogenization. In order to check the effect of emulsifier concentration on drug release and supersaturation, we prepared emulsions with 1 and 10 wt% emulsifier in the aqueous phase via rotor–stator homogenization. Sodium benzoate (preservative) and sodium chloride were dissolved in the aqueous solution of the emulsifier at concentrations of 2.5 and 0.06 wt%, respectively, for all emulsions studied.

The drug-loaded oily phase was prepared by dissolving fenofibrate at a concentration of 40 mg/mL in the studied fats and oils. The fats that are solids at room temperature (CB and CNO) were melted at *T* = 40 °C before the drug was dissolved.

#### 2.2.2. Emulsification Protocols

All emulsions were prepared at an oil volume fraction of 0.6. Emulsification was carried out at room temperature for the liquid oils (SFO and MCT) or at *T* = 40 °C for the solid fats (CB and CNO). After preparation, the emulsions were stored at room temperature for the liquid oils or at *T* = 40 °C for the solid fats.

For preparation of emulsions with bigger droplet size, we used rotor–stator homogenization via UltraTurrax T25 (IKA, Staufen, Germany) at 13,500 rpm for 3 min or sonication via SKL650-IIDN (Ningbo haishu sklon develop Ltd., Ningbo, China) at 1.0/0.5 s sonication/rest cycle at 350 W and total sonication time of 5 min. Nanoemulsions were prepared by high pressure homogenization (PandaPLUS 2000, GEA, Düsseldorf, Germany) at 500 bar and 10 passes.

#### 2.2.3. Emulsion Droplet Size Determination by Laser Diffraction

The droplet size of the prepared emulsions was characterized by Analysette 22 (Fritch, Germany), equipped with wet dispersing unit and working at a laser wavelength of 532 nm. The volume-weighted mean diameter (*d*_43_) was used as a measure for the emulsion droplet size. Each sample was measured in triplicate.

### 2.3. In Vitro Digestion Model

Lipid digestion and drug release were studied by an in vitro model of the gastrointestinal tract, which was previously used by our group to obtain useful mechanistic information about both the mechanisms of fenofibrate solubilization by polar lipids in biorelevant media [22] and about the impact of food components on lipid absorption [70,71,72]. The model consists of a gastric and a small intestinal stage, in which the enzymatic hydrolysis is represented by pepsin and pancreatin, and the intestinal pH is determined by a bicarbonate buffer. The concentration of bile salts used (10 mM) is representative for fed state conditions (post-prandial bile salt concentrations in human intestinal fluids are between 8 and 12 mM [73]). Calcium ions are also included in the model, as they have been shown to influence the solubilization of hydrophobic drugs and cholesterol [72,74].

Fenofibrate emulsions (60 vol. % oil) were added to the gastric phase of the digestion model at a constant volume of 0.833 mL, yielding a concentration of 1.33 mg/mL fenofibrate in the gastric phase. The switch to intestinal conditions was accompanied by a 1:1 dilution, resulting in a final fenofibrate concentration of 0.67 mg/mL. Fenofibrate concentrations of this order of magnitude could be expected when considering a single dose of 200 or 300 mg and real-life water intake (sip or half a glass of water [75]). The concentrations of emulsifier at intestinal conditions were 0.01 or 0.11% for the emulsions prepared with 1 or 10% emulsifier, respectively. These concentrations of the polysorbate 20 emulsifier do not inhibit lipid digestion by the pancreatic lipase, as they are significantly lower than the threshold inhibition concentrations reported in the literature (0.5 [76] and 1.2% [77]).

**Figure 1:**
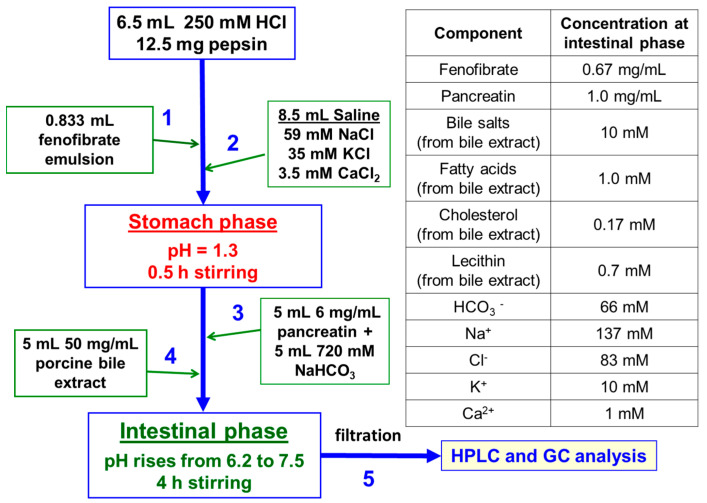
Schematic representation of the two-stage in vitro digestion model. Inset (right hand side): concentrations of the components at the intestinal phase.

The gastric phase was prepared by mixing 8.5 mL saline solution (59 mM NaCl, 35 mM KCl, 3.5 mM CaCl_2_) with 6.5 mL 0.25 M HCl in a glass bottle with pre-weighted pepsin (12.5 mg), yielding pH = 1.3. Then, the sample was stirred for 30 min. The shift from gastric to intestinal conditions was initiated by the sequential addition of 5 mL NaHCO_3_ (0.72 M), 5 mL porcine bile extract (50 mg/mL bile extract, pre-dissolved for 30 min at *T* = 37 °C) and 5 mL pancreatin (6 mg/mL), to obtain a final volume of 30 mL. The bottle was covered with a homemade glass cover and Teflon tape, on top of which the bottle cap was tightened and the samples were stirred. During the intestinal phase, the pH increased gradually from 6.2 to 7.5 after 240 min (due to release of CO_2_), mimicking the in vivo situation [78]. At the end of experiment, the samples were filtered with 1 µm cellulose filter followed by 200 nm NYLON filter. All experiments were performed at least in triplicate (*n* ≥ 3). The main steps in the in vitro digestion model and the concentration of the components in the intestinal stage are depicted in Figure 1.

### 2.4. Analytical Protocols

#### 2.4.1. Fenofibrate Determination by HPLC

After the end of the in vitro digestion experiment, 100 µL filtrate was diluted with 900 µL of 75:25 methanol:water and 20 μL was injected for HPLC analysis. The analysis was carried out on a Shimadzu apparatus, equipped with two high-pressure-mixing binary gradient pumps (LC-20AD), autosampler (SIL-10ADvp), four-line membrane degasser (DGU-14A), wide temperature range column oven (CTO-10ASvp) and a dual wavelength UV–VIS detector (SPD-10Avp). We used Waters X-bridge C18 analytical column (150 mm × 4.6 mm, 3.5 µm), connected to a Waters VanGard C18 guard column (3.9 mm × 5 mm, 3.5 µm). The mobile phase was methanol:water at a volume ratio of 75:25. The eluent flow rate was 1 mL/min and the column temperature was set at 40 °C. The retention time of fenofibrate was 10.1 min. The concentration of fenofibrate was determined by using a calibration curve (*R*^2^ = 0.999), which was prepared by dissolving a known amount of drug in methanol (see Figure S1 in the Supplementary Materials).

#### 2.4.2. Lipid Extraction and Analysis by GC

TG and their lipolysis products (FA, MG and DG) were determined by liquid–liquid extraction, followed by derivatization and GC, as adapted from Vinarova et al. [72]. Briefly, the protocol consisted of the following:

The pH of the filtrate obtained at the end of the in vitro digestion study was decreased to ≈2 by adding HCl to lower the solubility of the FA. Afterwards, chloroform was added (at a ratio of 1.5:2.0 chloroform:aqueous phase) and the sample was sonicated in a standard ultrasound bath for 15 min. The sample was homogenized by shaking after each 5 min of sonication. The extraction was completed by centrifugation of the obtained turbid dispersion for 30 min at 3622× *g*, leading to clear chloroform and aqueous phases.

The obtained organic solvent extracts were derivatized in the following way: 400 μL of the sample was mixed with 200 μL anhydrous pyridine, 100 μL internal standard (18 mg/mL hexadecanol in chloroform) and 200 μL BSTFA. The final volume of the derivatization mixture was 900 μL and the concentrations of the components were 56 vol. % chloroform, 22 vol. % pyridine, 22 vol. % BSTFA and 2 mg/mL hexadecanol. Then, the derivatization mixture was heated for 1 h at 60 °C. After cooling to room temperature, we diluted 75 μL of the derivatized sample with 950 μL isooctane. The instrument used for analysis was Agilent 8890 (Agilent technologies, Santa Clara, CA, USA), which was connected to autosampler 7693A. Agilent DB-5HT capillary column with the following specifications was used: (5%-Phenyl)-methylpolysiloxane, 30 m length, I.D. 0.32 mm, 0.1 μm film thickness. An injection volume of 1 μL and cold on-column injection was used. The oven was programmed in the following way: start at 60 °C, hold 1 min, the 1st ramp is to 180 °C at 10 °C/min, hold 0 min, 2nd ramp is to 375 °C at 30 °C/min, hold 10 min. Sample analysis time was 29.5 min. The flame ionization detector was operated at *T* = 380 °C. Helium at a constant flow of 2 mL/min was used as a carrier gas. Hydrogen, air and nitrogen (make-up gas) were used as detector gases.

The concentrations of FA, MG, DG and TG were calculated from the internal standard hexadecanol, using correction factors determined from calibration curves with standard substances. Retention times relative to the internal standard were used for peak identification (see Table S1 in the Supplementary Materials).

### 2.5. Extent of Lipid Digestion

Lipid digestion can be quantified by using the concentrations of the initial reactant (TG), or the reaction products (FA, MG, DG) in the sample. However, the filtration of the samples removes solids (e.g., insoluble or precipitated FA) and any oily phase (undigested TG). Thus, only a reaction product which is completely solubilized (thus is not removed during filtration) can be used to quantify lipid digestion. In a previous study performed by the same in vitro digestion model, we showed that examples of such products are the MG obtained after lipolysis of the long chain TG in SFO and CB [72]. A dedicated experiment with MCT confirmed that this trend is also valid for the shorter chain MCT oil: solubilization of 99.8% ± 8.1, which was measured for the C8 MG and C10 MG that were generated during in vitro digestion of MCT (determined by analyzing a sample before and after filtration). Therefore, the MG measured after filtration, *C*_MG_, was used to calculate the extent of TG hydrolysis to MG from the ratio *C*_MG_/*C*_TGini_, where *C*_TGini_ is the initial concentration of TG introduced in the digestion model (*C*_TGini_ was calculated based on the average molecular weight of TG, see Table 1).

### 2.6. Supersaturation Assessment

In order to determine if supersaturation takes place, we checked if the measured aqueous drug concentration exceeds the solubilization capacity of the medium. However, the lipolysis products generated during the in vitro digestion experiment continuously change the solubilization capacity of the simulated intestinal fluids [22]. Therefore, we used the following approaches.

The solubilization capacity of each SFO and CB digest was estimated by (1) determining the concentration of the lipolysis products by GC and (2) by taking into account the contribution of the generated oleic acid, linoleic acid, glycerol monooleate, glycerol monolinoleate, diolein and dilinolein to the overall solubilization capacity of the digest. The long chain saturated species generated during CB lipolysis were not taken into account, as they do not have a concentration-dependent effect on the solubilization capacity [22,72]. The used individual solubilization capacities, presented in units of µg/mL fenofibrate per mM of lipid, were calculated based on the data for fenofibrate solubilization by polar lipids in biorelevant media that we had obtained in a previous study, using the same in vitro digestion model [22]: 5.3 (oleic acid), 6.3 (linoleic acid), 7.5 (glycerol monooleate), 8.9 (glycerol monolinoleate), 12.7 (diolein) and 15.0 (dilinolein). The details of the calculation are presented in the Supplementary Materials.

In the case of MCT, we could not calculate the solubilization capacity of each digest, due to insufficient data about the individual contribution of their lipolysis products. To check for precipitation and determine the maximum fenofibrate solubilization, we added preservatives to the filtered MCT digests (which already contain orlistat), and we kept them for 15 days at *T* = 37 °C to ensure that equilibrium had been reached. Afterwards, the samples were filtered (to remove the formed precipitates), and the fenofibrate in the aqueous phase was measured again.

## 3. Experimental Results

We first present the droplet size of the prepared emulsions (Section 3.1). Afterwards, we consecutively describe the effect of lipid type (Section 3.2), droplet size and surfactant concentration (Section 3.3) on TG lipolysis and fenofibrate release. Finally, the assessment of drug supersaturation and the impact of the generated lipid digestion products on fenofibrate solubilization are presented (Section 3.4).

### 3.1. Emulsion Droplet Size

We prepared a total of 10 emulsions from four lipid carriers with pre-dissolved fenofibrate (see Section 2.2 for more details). The emulsion drop size was characterized by laser diffraction and the results are presented in Table 2. A set of nanoemulsions (*d*_43_ < 1 µm), fine emulsions (*d*_43_ ≈ 2 µm) and coarse emulsions (*d*_43_ > 9 µm) were obtained for SFO and MCT, providing a sufficient spread to evaluate the impact of droplet size on drug release and lipid digestion. SFO and MCT oils were selected to study the impact of droplet size, due to their very different digestion and drug release profiles; see Section 3.2. At the same time, emulsions with the four lipid carriers with a similar drop size were obtained (*d*_43_ from 1.4 to 2.2 µm), allowing us to study the effect of the lipid type on drug release at similar initial conditions.

### 3.2. Effect of Lipid Type on Lipolysis and Fenofibrate Release

**Figure 2:**
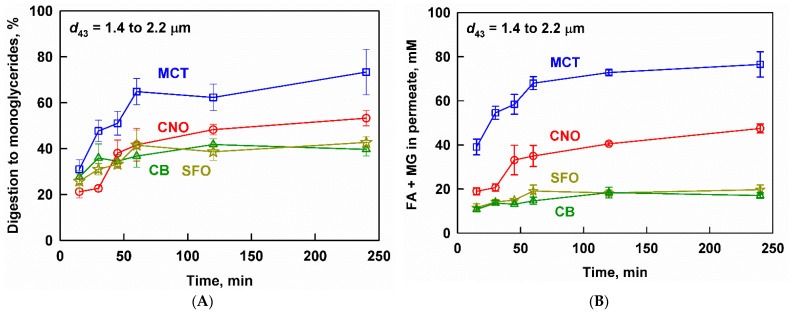
(**A**) Extent of lipid digestion to monoglycerides and (**B**) fatty acids (FA) and monoglycerides (MG) measured in the aqueous phase after filtration, as a function of the digestion time at simulated intestinal conditions of MCT (blue squares), CNO (red circles), SFO (yellow stars) and CB (green triangles) emulsions (*d*_43_ between 1.4 and 2.2 µm). Emulsifier concentration = 1 wt% for all emulsions.

In this section, we describe the lipolysis and drug release from fenofibrate-loaded lipid emulsions prepared from four lipid carriers (MCT, CNO, SFO and CB), which were characterized by a similar droplet size (*d*_43_ from 1.4 to 2.2 µm). As lipid digestion can trigger drug release by hydrolyzing the TG-based carrier and/or by increasing the drug solubilization capacity of the biorelevant media, we first describe the extent of lipid digestion to MG; see Figure 2A.

MCT was digested to a larger extent (73%) compared to the long chain CNO (53%), SFO (43%) and CB (40%). The overall kinetics of the process were similar in the studied fats and oils, in that most of the digestion occurred in the first 60 min of the experiment. The measured extent of the digestion to MG can also be used to assess whether or not an oily phase composed of TG was still intact at the end of the experiments. In our previous work, we found that a degree of digestion to MG of 60% corresponds to the complete digestion of the TG, by using the same in vitro model [72]. Hence, we can suggest that the TG in the MCT formulations have been completely digested, whereas a small fraction of undigested TG is most likely present after the digestion of the CNO, SFO and CB emulsions.

Lipid digestion products, such as FA and MG, are also released in the aqueous phase during the lipolysis of the carrier lipids; see Figure 2B. For MCT, CNO and SFO, the kinetics of this process mirrored the digestion kinetics: two regions were observed, consisting of a significant initial increase of the digestion products (in the first 45–60 min of the digestion), followed by a slower increase of up to 240 min (for the short-chain lipids) or a plateau (for SFO). For CB, the concentration of digestion products in the aqueous phase slowly increased throughout the experiment.

However, significant quantitative differences in the concentrations of FA+MG were observed depending on the lipid type: the highest concentrations of FA+MG were measured for MCT (39–77 mM), followed by CNO (19–48 mM), whereas the concentrations for SFO and CB were much lower (11–20 mM). These differences are explained by (1) the extent of digestion (Figure 2A) and (2) the experimental design of the study, where all lipid emulsions were introduced into the in vitro digestion model at an equal *mass*, resulting in different *molar* concentrations of TG (due to the molecular weight differences, where SFO ≈ CB > CNO > MCT, see Table 1).

**Figure 3:**
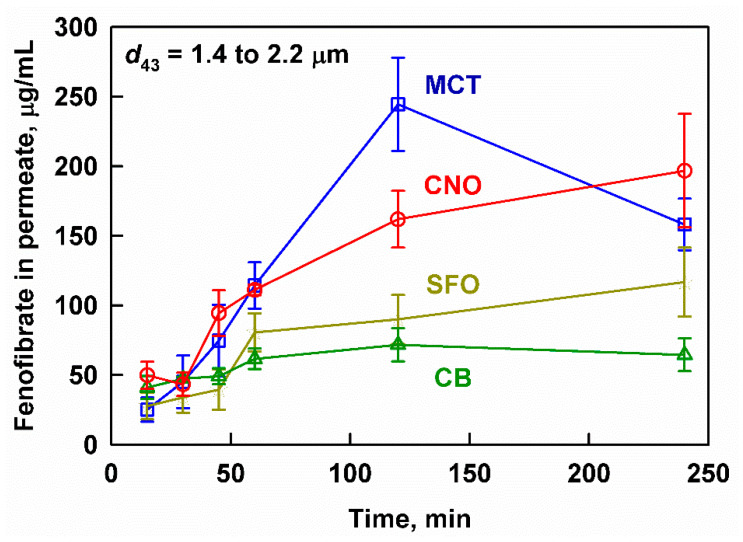
Fenofibrate measured in the aqueous phase after filtration as a function of the digestion time at simulated intestinal conditions of MCT (blue squares), CNO (red circles), SFO (yellow stars) and CB (green triangles) emulsions prepared by sonication (*d*_43_ between 1.4 and 2.2 µm). Emulsifier concentration = 1 wt% for all emulsions.

The measured concentration of fenofibrate in the aqueous phase is presented as a function of digestion time in Figure 3. The highest drug concentrations were measured for MCT, followed by CNO, SFO and CB. A lag-time in the drug release was observed, which was manifested in the similar and relatively low concentrations of fenofibrate (25 ± 9 to 50 ± 10 µg/mL) for all studied lipid carriers in the first 30 min of digestion. As digestion progressed, the fenofibrate release increased significantly for MCT, reaching a maximum of 245 ± 34 µg/mL after 120 min, which decreased to 158 ± 19 µg/mL after 240 min. The other lipid carriers exhibited a monotonic drug release, reaching maximum fenofibrate concentrations after 240 min: 197 ± 41, 117 ± 25 and 64 ± 12 µg/mL fenofibrate for CNO, SFO and CB, respectively. All measured concentrations were significantly higher than the solubility of fenofibrate in blank media (without oil emulsion or enzymes), which was determined to be 6.5 ± 0.5 µg/mL in our previous study [22]. Digestion was key to initiating the drug release from the drug-loaded fenofibrate emulsions: the aqueous fenofibrate concentrations in control experiments without enzymes were below the detection limit.

### 3.3. Effect of Drop Size and Surfactant Concentration on Lipolysis and Fenofibrate Release

In order to study the effect of emulsion drop size on drug release and lipid digestion, we selected the oil with the highest degree of digestion and aqueous fenofibrate concentrations (MCT) and one with incomplete digestion and lower drug concentrations (SFO). In order to determine the effect of the emulsion drop size, we complemented the existing data (*d*_43_ = 1.9–2.2 µm) by studying emulsions with a smaller (*d*_43_ = 0.4–0.7 µm) and larger drop size (*d*_43_ = 11.8–13.9 µm). The effect of the surfactant concentration was studied at 1 and 10% surfactant.

#### 3.3.1. Sunflower Oil Emulsions

**Figure 4:**
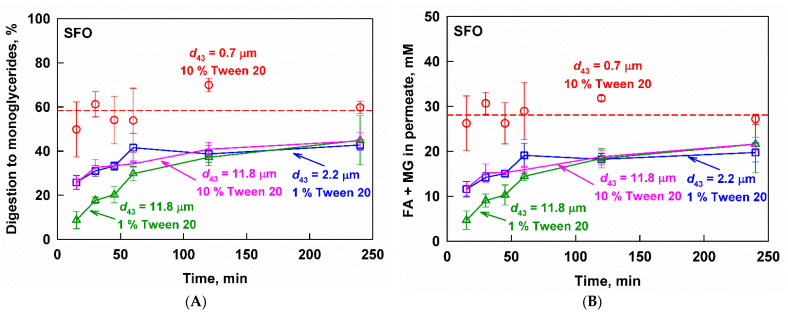
(**A**) Digestion and (**B**) fatty acids (FA) and monoglycerides (MG) measured in the aqueous phase after filtration of fenofibrate-loaded SFO emulsions with *d*_43_ = 0.7 µm (10 wt% emulsifier, red circles), *d*_43_ = 2.2 µm (1 wt% emulsifier, blue squares), *d*_43_ = 11.8 µm (10 wt% emulsifier, pink stars) and *d*_43_ = 11.8 µm (1 wt% emulsifier green triangles) as a function of time at simulated intestinal conditions.

The effect of drop size on the digestion to MG of the fenofibrate-loaded SFO emulsions is presented in Figure 4A. Digestion was slowest for the emulsion with the largest drop size (11.8 µm) and low surfactant concentration (1%), reaching only 9 ± 4% in the first 15 min of in vitro intestinal digestion. Decreasing the drop size to 2.2 µm or increasing the surfactant concentration to 10% significantly increased the digestion rate (26 ± 3% digestion at 15 min). A further decrease in the drop size led to a very quick digestion of the nanoemulsion (0.7 µm), reaching a plateau of 59 ± 13% at *t* = 15 min. While the digestion of the nanoemulsion did not progress any further, the digestion of the emulsions with a bigger drop size gradually increased up to 45 ± 11%.

As expected, the concentration of lipid digestion products (FA+MG) in the aqueous phase was a mirror image of the digestion kinetics (Figure 4B): the nanoemulsion generated very high FA+MG concentrations (28 ± 4 mM) in the first 15 min of the reaction, which remained constant up to 240 min. On the other hand, the emulsions with a larger drop size generated much lower concentrations of digestion products, which gradually increased with time.

In agreement with the digestion kinetics, increasing the surfactant concentration from 1 to 10 wt% for the emulsion with *d*_43_ = 11.8 µm significantly increased the rate of reaction products generation and shifted the FA+MG profile identical to the 2.2 µm emulsion.

**Figure 5:**
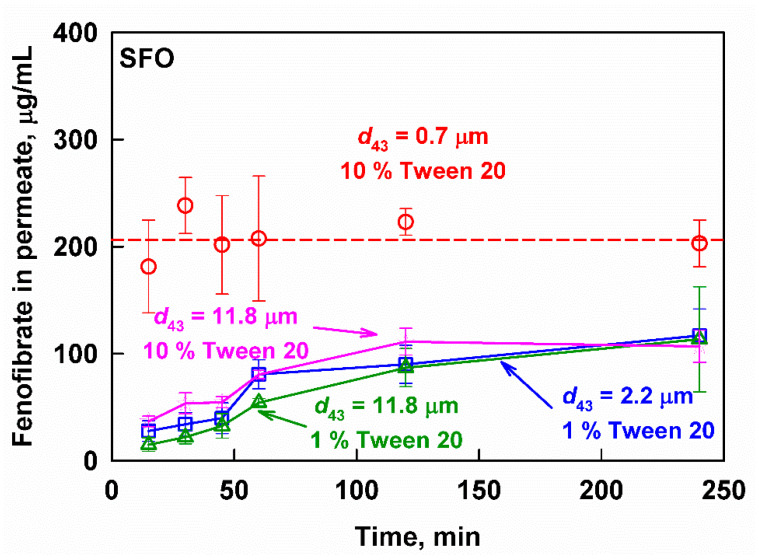
Fenofibrate measured in the aqueous phase after filtration as a function of the digestion time at simulated intestinal conditions of SFO emulsions with *d*_43_ = 0.7 µm (10 wt% emulsifier, red circles), *d*_43_ = 2.2 µm (1 wt% emulsifier, blue squares), *d*_43_ = 11.8 µm (10 wt% emulsifier, pink stars) and *d*_43_ = 11.8 µm (1 wt% emulsifier green triangles).

The fenofibrate release from the SFO formulations was also affected significantly by the emulsion drop size (Figure 5). An average concentration of 206 ± 38 µg/mL fenofibrate was reached very quickly for the nanoemulsion (*d*_43_ = 0.7 µm) and remained constant throughout the 240 min of the experiment. In contrast, fenofibrate was released slowly from the larger drop size emulsions (*d*_43_ = 2.2 and 11.8 µm), starting from below 30 µg/mL in the first 15 min of the experiment, and reaching 113 ± 49 µg/mL at 240 min.

Increasing the surfactant concentration from 1 to 10% for the 11.8 µm emulsion significantly increased the initial drug release rate (from 15 ± 6 to 37 ± 5 µg/mL fenofibrate, respectively). However, the aqueous fenofibrate concentrations remained much lower, compared to the ones measured after the digestion of the nanoemulsion (206 ± 38 µg/mL).

#### 3.3.2. Medium-Chain Triglyceride Emulsions

**Figure 6:**
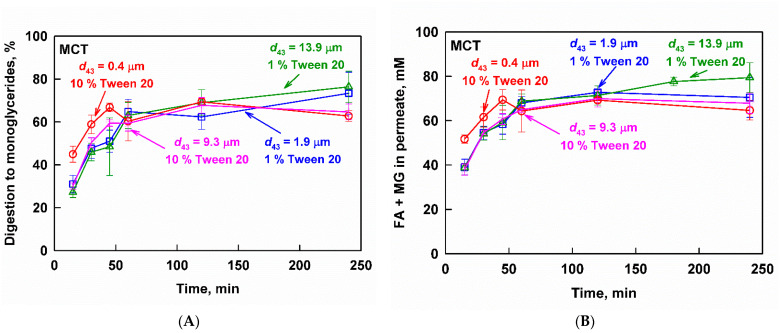
(**A**) Digestion and (**B**) fatty acids (FA) and monoglycerides (MG) measured in the aqueous phase after filtration of fenofibrate-loaded MCT emulsions with *d*_43_ = 0.4 µm (10 wt% emulsifier, red circles), *d*_43_ = 1.9 µm (1 wt% emulsifier, blue squares), *d*_43_ = 9.3 µm (10 wt% emulsifier, pink stars) and *d*_43_ = 13.9 µm (1 wt% emulsifier green triangles) as a function of time, at simulated intestinal conditions.

The effect of drop size on the digestion to MG of the shorter-chain-length MCT emulsions is presented in Figure 6A. The nanoemulsion (*d*_43_ = 0.4 µm) was digested slightly faster in the initial 45 min of the experiment, reaching 67 ± 2% digestion, compared to the larger drop size emulsions (*d*_43_ = 1.9, 0.3 and 13.9 µm), which were digested more slowly (≈50% digestion at *t* = 45 min). The emulsion with a drop size of 9.3 µm and surfactant concentration of 10% showed a similar digestion profile to the emulsions prepared with 1% surfactant and a drop size in the range between 1.9 and 13.9 µm. Hence, the surfactant concentration did not alter the digestion of the MCT emulsions.

No significant differences in the digestion of the four emulsions were observed in the later stages of the experiment, where all emulsions reached a similar extent of digestion of 72.0 ± 10.0 at 240 min. The measured degrees of digestion to MG indicated a nearly complete digestion of the TG at the end of the experiment [72].

The measured concentration of FA+MG after filtration were in agreement with the results for the extent of digestion and followed the same trends (Figure 6B): slightly higher lipid digestion products concentrations were measured in the initial 45 min of the experiment for the nanoemulsion (compared to the larger drop size emulsions), whereas, at longer times, the different emulsion drop size did not produce any significant effects. The surfactant concentration did not have any significant effect as well.

**Figure 7:**
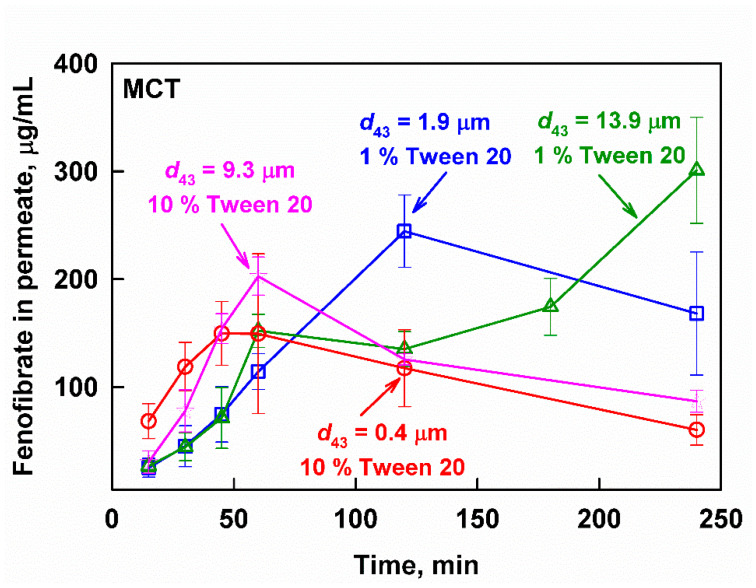
Fenofibrate measured in the aqueous phase after filtration as a function of the digestion time at simulated intestinal conditions of MCT emulsions with *d*_43_ = 0.4 µm (10 wt% emulsifier, red circles), *d*_43_ = 1.9 µm (1 wt% emulsifier, blue squares), *d*_43_ = 9.3 µm (10 wt% emulsifier, pink stars) and *d*_43_ = 13.9 µm (1 wt% emulsifier green triangles).

Vastly different kinetics of the fenofibrate release were observed depending on both the drop size and the surfactant concentration of the MCT emulsions; see Figure 7. The digestion of the nanoemulsion (*d*_43_ = 0.4 µm, 10% surfactant) resulted in an aqueous fenofibrate concentration profile that passed through a maximum at *t*_max_ = 45 min (*C*_max_ = 150 ± 30 µg/mL) and then decreased, indicating precipitation. Increasing the drop size to 9.3 µm at the same surfactant concentration of 10% resulted in a slower drug release (*t*_max_ = 60 min) and higher drug concentrations (*C*_max_ = 203 ± 18 µg/mL) before the onset of precipitation. Decreasing the surfactant concentration from 10 to 1% further increased the time required to reach maximum drug concentrations and the value at the maximum. For the 1.9 µm emulsion, *C*_max_ of 245 ± 34 µg/mL was measured at *t*_max_ = 120 min, whereas the 13.9 µm emulsion was characterized by a *C*_max_ = 301 ± 49 µg/mL at *t*_max_ = 240 min.

### 3.4. Interplay between Solubilization, Supersaturation and Drug Release

#### 3.4.1. Effect of Emulsion Drop Size

In this section, we further analyze the datasets which comprise SFO and MCT emulsions with different drop sizes and surfactant concentrations (their digestion and drug release were already presented in Section 3.3). In particular, we look for a correlation between the aqueous fenofibrate and the aqueous lipid digestion products and we evaluate if supersaturated drug concentrations are reached.

**Figure 8:**
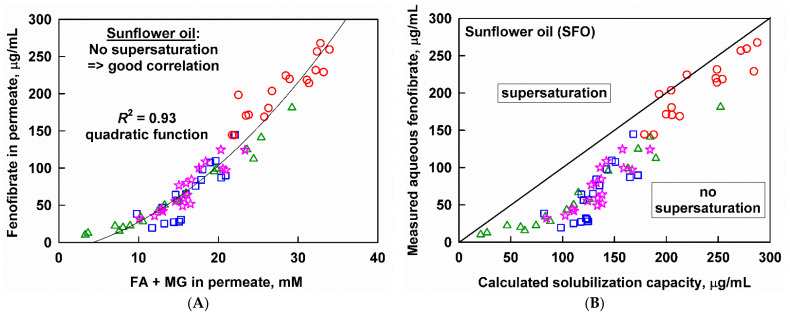
Aqueous fenofibrate as a function of (**A**) the sum of fatty acids (FA) and monoglycerides (MG) measured in the aqueous phase or (**B**) the calculated solubilization capacity, after digestion of SFO emulsions with *d*_43_ = 0.7 µm (10 wt% emulsifier, red circles), *d*_43_ = 2.2 µm (1 wt% emulsifier, blue squares), *d*_43_ = 11.8 µm (10 wt% emulsifier, pink stars) and *d*_43_ = 11.8 µm (1 wt% emulsifier green triangles). Individual (non-averaged) data points are plotted.

The aqueous fenofibrate concentrations measured after the in vitro lipolysis of the four SFO emulsions with different initial droplet sizes and different surfactant concentrations collapsed on the same master curve (quadratic function, *R*^2^ = 0.93) when plotted against the FA+MG in the aqueous phase (Figure 8A). Note that the plots on Figure 8 were constructed by using individual data points, rather than the averaged data.

In order to check if the released fenofibrate is in a supersaturated state, we compared the measured concentrations with the solubilization capacity of each respective digest, calculated as described in Section 2.6. It was found that fenofibrate is below or near the solubilization capacity of the media (Figure 8B); hence, no drug supersaturation was observed upon the in vitro digestion of the SFO-based fenofibrate formulations, regardless of the initial droplet size or the surfactant concentration.

Two peculiar features of Figure 8 deserve additional attention. First, a closer look at the correlation of solubilized fenofibrate with the soluble reaction products (Figure 8A) shows that the non-linear part comprises data points obtained from the larger drop size emulsions (*d*_43_ = 2.2 and 11.8 µm). The same data points are also below the calculated fenofibrate solubilization capacity (Figure 8B). The last observation could be explained if a kinetically limited (viz. time-dependent) process plays a role in the transfer of fenofibrate to the aqueous phase.

**Figure 9:**
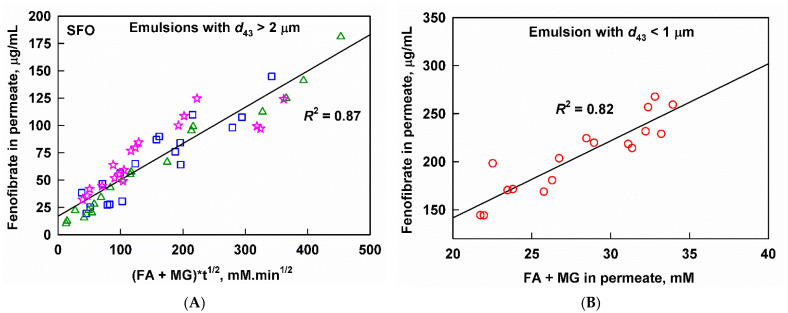
Aqueous fenofibrate as a function of (**A**) the sum of fatty acids (FA) and monoglycerides (MG) measured in the aqueous phase multiplied by *t*^1/2^ for microscale SFO emulsions with *d*_43_ = 0.7 µm (10 wt% emulsifier, red circles), *d*_43_ = 2.2 µm (1 wt% emulsifier, blue squares) and *d*_43_ = 11.8 µm (10 wt% emulsifier, pink stars), and (**B**) the sum of fatty acids (FA) and monoglycerides (MG) measured in the aqueous phase for the nanoscale SFO emulsion with *d*_43_ = 0.7 µm (red circles). Individual (non-averaged) data points are plotted.

In order to check if this is the case, we introduced *time* as an additional variable in the correlation; see Figure 9A. As a result, a relatively good linear correlation (*R*^2^ = 0.87) of solubilized fenofibrate with the digestion products (FA+MG) and time (*t*^1/2^) was observed for the large drop size (*d*_43_ = 2.2 and 11.8 µm) SFO emulsions. For the SFO nanoemulsion (*d*_43_ = 0.7 µm), a linear correlation (*R*^2^ = 0.82) was observed by using only the concentration of the digestion products, indicating that the drug transfer from the oil droplets to the colloidal pseudo phase was not kinetically limited in this case (Figure 9B). We also attempted to correlate the aqueous fenofibrate to single lipid digestion product concentration or different combinations of the digestion products, but the obtained *R*^2^ was lower than 0.80. The physical meaning and mechanistic understanding that can be extracted from these correlations are discussed in Section 4.1.

The effect of droplet size and surfactant concentration on drug release from the MCT-based fenofibrate formulations can be analyzed in a similar way. Hence, we must first clarify whether drug supersaturation is observed, or if the measured concentrations can be explained by solubilization. We determined the equilibrium fenofibrate solubilization in selected digests, obtained after 60 min (for the nanoemulsion, *d*_43_ = 0.4 µm), 120 min (for the emulsion with *d*_43_ = 1.9 µm) or after 240 min (for the emulsion with *d*_43_ = 13.9 µm) in in vitro digestion. These digestion times were selected in order to reproduce the maximum aqueous fenofibrate concentrations obtained after the digestion of the respective emulsions (Figure 7). In all cases, we observed precipitation, leading to a decrease of the aqueous fenofibrate concentration; the measured equilibrium fenofibrate solubilization capacity for all three digests was 131 ± 20 µg/mL (see Figure S2 in the Supplementary Materials) and this value was used as a reference for drug supersaturation.

**Figure 10:**
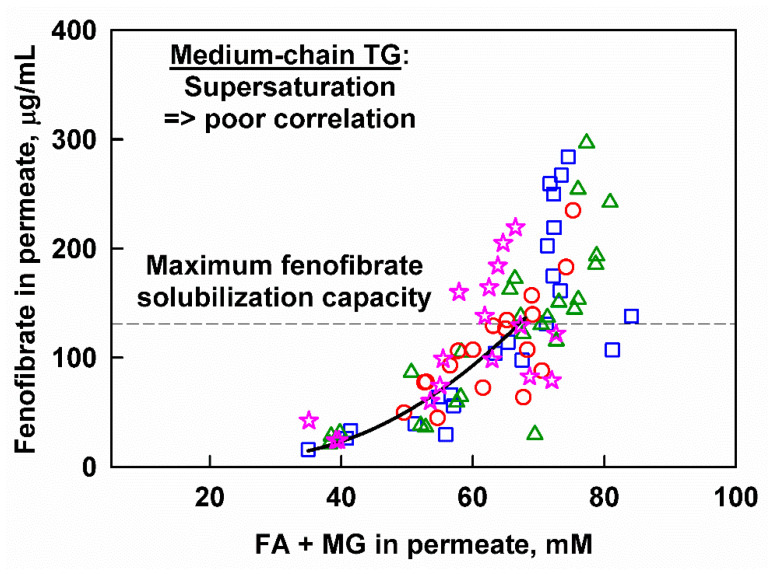
Aqueous fenofibrate as a function of the sum of fatty acids (FA) and monoglycerides (MG) measured in the aqueous phase after in vitro digestion of MCT emulsions with *d*_43_ = 0.4 µm (10 wt% emulsifier, red circles), *d*_43_ = 1.9 µm (1 wt% emulsifier, blue squares), *d*_43_ = 9.3 µm (10 wt% emulsifier, pink stars) and *d*_43_ = 13.9 µm (1 wt% emulsifier green triangles).

The concentration of released fenofibrate from the studied MCT emulsions is plotted as a function of the lipid digestion products in Figure 10. The same general trend was observed: a gradual increase of released fenofibrate with the increase of lipid digestion product concentrations is observed at concentrations below the solubilization limit, whereas a sharp increase that does not correlate with the digestion products is observed for the supersaturated drug concentrations.

#### 3.4.2. Effect of Lipid Carrier Type

**Figure 11:**
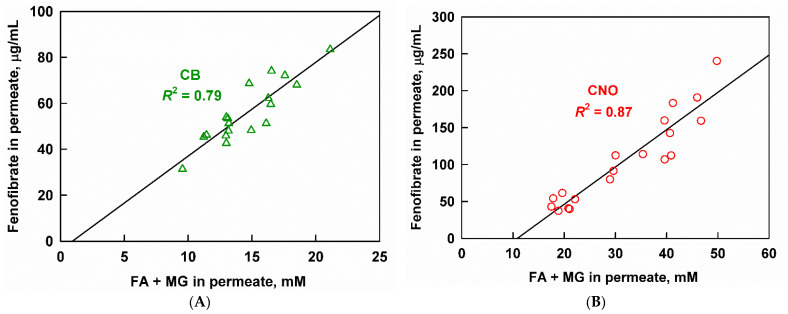
Aqueous fenofibrate as a function of the sum of fatty acids (FA) and monoglycerides (MG) measured in the aqueous phase after in vitro digestion of (**A**) cocoa butter and (**B**) coconut oil emulsions.

The results presented in the previous section showed that aqueous drug concentrations correlate well with the solubilized lipid digestion products for non-supersaturated MCT and SFO digests. While MCT and SFO represent medium-chain and unsaturated oils (two of the most widely used classes), they do not cover the chemical space of long-chain saturated fats and oils (which are abundant in nature). Hence, the aqueous fenofibrate concentrations obtained after the digestion of CB and CNO-based formulations were plotted as a function of the aqueous FA and MG concentrations; see Figure 11.

Relatively good linear correlations were observed for CB (*R*^2^ = 0.79) and CNO (*R*^2^ = 0.87) when the sum of all FA and MG was used. Worse correlations were obtained when only the unsaturated lipid digestion products were used, indicating that the saturated species may also play some role in drug solubilization (see Figure S3 in the Supplementary Materials). For CB, the measured aqueous fenofibrate was below or near the calculated solubilization capacity of the digest, indicating that supersaturation was not achieved for this system (see Figure S4 in the Supplementary Materials). For CNO, we could not calculate the solubilization capacity of the digests in order to check for supersaturation; however, the good correlation of the aqueous fenofibrate with the sum of FA and MG suggests that supersaturation is not achieved in this case as well (this type of correlation deteriorates in cases of supersaturation, as shown for MCT in the previous section).

## 4. Discussion

### 4.1. Drug Solubilization and the Role of Lipid Digestion Products

In the current study, we investigated the drug release from MCT, SFO, CB and CNO-based oil-in-water emulsions with initial droplet sizes in the nano- and micrometer range. We found a good, generally linear correlation between aqueous fenofibrate and the sum of FA+MG concentrations in the aqueous phase for all systems (Section 3.4), except for the completely digested MCT emulsions, where supersaturation was observed (discussed in the following Section 4.2).

An interesting behavior was displayed by the larger drop size SFO emulsions: a non-linear relation between aqueous fenofibrate and aqueous FA+MG was established (Figure 8A), which was linearized by introducing √*t* as an additional independent variable (Figure 9A), yielding a good correlation (*R*^2^ = 0.87). From a mechanistic viewpoint, the dependence on √*t* can be explained in several ways.

For example, the diffusion of fenofibrate molecules from the bulk of the oil droplet to its surface (where transport to the aqueous phase takes place) can be the rate-limiting step in fenofibrate solubilization, in the case where the rate of increase in the solubilization capacity of the aqueous phase (driven by the generation of digestion products) is very fast. This explanation is in line with several experimental observations: (1) the aqueous fenofibrate concentrations measured during the digestion of the larger drop size emulsions are lower than the calculated solubilization capacity of corresponding digests (Figure 8B), (2) the (FA+MG) * √*t* expression does not work for the SFO nanoemulsion, which has a much higher surface-to-volume ratio and (3) the phenomena is not observed upon the digestion of emulsions prepared with a shorter chain (MCT) or saturated fats and oils (CB, CNO), which generate lipolysis products with a lower solubilization capacity (long-chain unsaturated lipids have a much higher solubilization capacity than shorter-chain or saturated lipids [22,72]).

An alternative hypothesis can be constructed if we focus on the solubilizing capacity of the oily phase, in which the fenofibrate is administered. A control experiment in the absence of enzymes (*viz*. no lipid digestion) showed practically zero drug release when the fenofibrate-loaded SFO emulsion was not digested. The latter example illustrates that the transfer of fenofibrate to the aqueous phase is an energetically unfavorable process (due to the high solubility of fenofibrate in SFO), which can only be forced by the digestion of the lipid phase. We can then suggest that the slow digestion (from 9 to 41% in 2 h) of large-drop-size (>2 µm) SFO emulsions can explain the measured low fenofibrate solubilization for these systems (see Figure 8B). Therefore, lipid digestion might be the rate-limiting process that underpins the √*t* dependence of fenofibrate solubilization.

However, it should be noted that both hypotheses consider the competition between two processes (lipolysis and drug transfer from oil to aqueous phase) whose rate might depend on the hydrodynamic conditions to a different extent. Hence, one should be careful when extrapolating from the in vivo situation or even from in vitro studies performed at different conditions.

### 4.2. Supersaturation and Precipitation

At gastrointestinal conditions, the supersaturation of lipid formulations is generally governed by their composition: for example, surfactant- and cosolvent-rich formulations (type III and IV) usually form microemulsions in aqueous media, leading to rapid drug release and supersaturated drug concentrations right after dispersion (before lipolysis has been initiated) [79,80]. In line with the observations in the current paper, supersaturation was not observed upon the digestion of type I or II lipid formulations based on long-chain TG [42].

In contrast, the digestion of MCT-based formulations was usually associated with increased supersaturation in the literature [42]. In the current study, the aqueous fenofibrate concentrations increased gradually as digestion progressed, until a critical point where triggered supersaturation was reached (Figure 10). Further analysis of the data showed that this critical point corresponds to an extent of digestion to MG of 60% (see Figure S5 in the Supplementary Materials), which indicates a nearly complete digestion of the oily TG phase [72]. Hence, the complete lipolysis of the studied MCT formulations appeared to trigger fenofibrate release and supersaturation, in agreement with the mechanism proposed in the literature [42]. Recently, this was also illustrated with type III and IV fenofibrate formulations that were studied in an in vitro model of rat digestion, which showed a good correlation with fenofibrate absorption in rats [80].

Since supersaturation is a transient, unstable state, it is logical to expect an effect of the emulsion droplet size or surfactant concentration. Indeed, both factors were found to play a role. A lower surfactant concentration and larger drop size decreased the speed at which peak supersaturation was reached, but also promoted higher fenofibrate concentrations at the peak. For example, the time required to reach the maximum fenofibrate concentration (*t*_max_) correlates very well (*R*^2^ = 0.996) with the logarithm of the emulsion droplet size (see Figure S6 in the Supplementary Materials): the smaller the droplet size, the less time it takes to reach supersaturated fenofibrate concentrations. This could be rationalized, considering that the higher surface-to-volume ratio of finer emulsions will accelerate both the lipolysis (as seen in Figure 6A) and the mass transfer of the drug from the oily to the aqueous phase.

Therefore, a smaller drop size and fast drug release might not necessarily be better as far as MCT-based type I lipid formulations are considered. However, these kinetic considerations should be extrapolated with caution to the in vivo situation, where aqueous drug concentrations could be strongly affected by permeation (especially for highly permeable drugs, such as fenofibrate), hence altering the supersaturation profile.

## 5. Conclusions

We studied the impact of droplet size (*d*_43_ from 0.4 to 13.9 µm), surfactant concentration (1 and 10% Tween 20) and lipid carrier type (MCT, SFO, CNO and CB) on fenofibrate solubilization and supersaturation during the in vitro digestion of type I lipid formulations. Short-chain triglycerides were key in promoting faster drug release: MCT > CNO > SFO ≥ CB. Fenofibrate release from long-chain triglyceride formulations (SFO, CNO and CB) was governed by solubilization and was enhanced at a smaller droplet size and higher surfactant concentration. Aquesous fenofibrate concentrations correlated well with the lipid digestion products (FA+MG) in the aqueous phase of these digests, illustrating the solubilization enhancement effect of the generated lipolysis products. In contrast, supersaturation was observed for MCT emulsions due to two factors: (1) the complete triglyceride digestion, which effectively expelled the fenofibrate from the lipid phase and (2) the low solubilization capacity of the medium-chain lipolysis products. A smaller drop size and higher surfactant concentration had negative effects on fenofibrate supersaturation: lower peak fenofibrate concentrations and a faster onset of precipitation were observed.

The used experimental approach provides useful mechanistic information for the impact of droplet size and surfactant concentration on drug supersaturation and solubilization after the in vitro digestion of type I lipid formulations, and could potentially be applied to other types of LBF. The good correlation of drug solubilization with the concentrations of lipolysis products suggests that the in silico prediction of lipid-digestion-driven solubilization effects (whether in the fed state or after the ingestion of lipid formulations) may be possible in the future.

## Figures and Tables

**Table 1:** Fatty acid profile and average triglyceride molecular weight of the studied lipids.

	FA Profile of the Studied Lipid Carriers (%)			
FA	MCT	CNO	CB	SFO
**C8:0**	71.0	6.6	*n.d.*	*n.d.*
**C10:0**	29.0	5.6	*n.d.*	*n.d.*
**C12:0**	*n.d.*	48.0	*n.d.*	*n.d.*
**C14:0**	*n.d.*	18.8	*n.d.*	*n.d.*
**C16:0**	*n.d.*	9.5	26.6	7.1
**C18:0**	*n.d.*	3.4	38.6	5.7
**C18:1,2**	*n.d.*	8.0	34.8	87.2
**average molecular weight, g/mol**	495	682	866	876

*n*.*d.* = not detected.

**Table 2:** Drop size of the prepared fenofibrate-loaded emulsions, presented as the volume-weighted mean diameter, *d*_43_. The number of emulsions prepared and used in the study is denoted as *n* (*n* = 1 indicates that the same emulsion was used for all experiments in the study).

Emulsification Equipment	Lipid Type	Tween 20, wt%	*d*_43_, µm		
			AVG	SD	*n*
High-pressure homogenizer	SFO	10	0.7		1
Sonicator	SFO	1	2.2	0.1	2
Rotor–stator homogenizer	SFO	1	11.8	0.2	2
Rotor–stator homogenizer	SFO	10	11.8	0.8	2
High-pressure homogenizer	MCT	10	0.4	0.1	2
Sonicator	MCT	1	1.9	0.2	3
Rotor–stator homogenizer	MCT	1	13.9	1.0	2
Rotor–stator homogenizer	MCT	10	9.3	0.4	2
Sonicator	CNO	1	2.0		1
Sonicator	CB	1	1.4		1

## Data Availability

The data that support the findings of this study are available from the corresponding author upon reasonable request.

## Supplementary Materials

The following are available online at https://www.mdpi.com/article/10.3390/pharmaceutics13081287/s1: procedure for determination of the fatty acid profile of the studied fats and oils; calculation of the solubilization capacity of the SFO digests; Table S1, relative retention times (RRT) of lipids used for peak identification in GC analysis; Table S2, solubilization capacities of the SFO lipolysis products; Figure S1. used for quantification of fenofibrate by HPLC-UV; Figure S2. Aqueous fenofibrate after digestion of MCT emulsions; Figure S3. Aqueous fenofibrate as a function of (A) the unsaturated monoglycerides and unsaturated fatty acids in the permeate or (B) the unsaturated monoglycerides, unsaturated fatty acids and the diglycerides in the permeate; Figure S4. Aqueous fenofibrate as a function of the calculated solubilization capacity after diges-tion of CB emulsions; Figure S5. Aqueous fenofibrate as a function of the digestion to monoglycerides measured after in vitro digestion of MCT emulsions; Figure S6. (A) Digestion time required to reach maximum aqueous fenofibrate concentrations (tmax) as a function of MCT emulsion droplet size, (B) maximum aqueous fenofibrate concentrations (Cmax) as a function of MCT emulsion droplet size; Figure S7. Supersaturation parameters after digestions of MCT emulsions: maximum fenofibrate concentration (Cmax) as a function of the time required to reach that concentration (tmax).
