# Supplementary material for: Supersaturation and Solubilization upon In Vitro Digestion of Fenofibrate Type I Lipid Formulations: Effect of Droplet Size, Surfactant Concentration and Lipid Type
Source: Pharmaceutics. 2021 Aug 18;13(8):1287. doi: 10.3390/pharmaceutics13081287 (PMC8399075; doi:10.3390/pharmaceutics13081287)
Supplement: Supplementary file 1 [file pharmaceutics-13-01287-s001.zip › pharmaceutics-1337307-supplementary.pdf]

pharmaceutics

Supplementary Materials: Supersaturation and Solubilization upon In Vitro Digestion of Fenofibrate Type I Lipid Formulations: Effect of Droplet Size, Surfactant Concentration and Lipid Type

Vladimir Katev, Sonya Tsibranska-Gyoreva, Zahari Vinarov and Slavka Tcholakova

Procedure for determination of the fatty acid profile of the studied fats and oils

To determine the fatty acid composition of the used fats and oils, they were saponified by the following procedure, adapted from IUPAC (Paquot, C. 1981. Standard methods for the analysis of oils, fats and derivatives (IUPAC), In *Pure & appl. Chem* (6th ed.)). First, we prepared 3.33 M alcoholic (80 % ethanol) solution of potassium hydroxide (KOH). Then, the fats and oils which are solids at room temperature (cocoa butter (CB), and coconut oil (CNO)) were melted at 50 ºC, 0.22 g of each were weighted in a separate vessel and 3 mL of the alc. KOH solution were added. The vessel was tightly closed and left for 4 hours in a heating oven set at 45 ± 5 ºC. The same procedure, but at room temperature, was applied to sunflower oil (SFO) and the medium chain triglycerides (MCT) as they are liquids. Every hour the mixture in the vessel was homogenized by hand shaking. Afterwards, the obtained clear solution was left to evaporate in a vacuum dryer overnight. The next day, 24.5 mL of water were added to the vessel and the pH was lowered to 2 by addition of 5.5 mL 2 M HCl, in order to convert the potassium fatty acid soaps to non-ionized fatty acids. The sample was then extracted with chloroform, derivatized and analyzed by GC.

Table S1. Relative retention times (RRT) of lipids used for peak identification in GC analysis, calculated *vs*. cetanol internal standard.

Compound		RRT	SD
FA	C8	2.241	0.034
FA	C10	1.621	0.018
FA	C12	1.285	0.007
FA	C14	1.074	0.012
MG	C8	1.066	0.007
FA	C16	0.965	0.003
MG	C10	0.963	0.000
FA	C18:1	0.915	0.001
FA	C18	0.908	0.006
MG	C12	0.907	0.000
MG	C14	0.868	0.000
MG	C16	0.862	0.000
MG	C18	0.832	0.010
MG	C18:1	0.824	0.000

Calculation of the solubilization capacity of the SFO digests

The individual fenofibrate solubilization capacity of oleic acid (OA), linoleic acid (LA) and glycerol monooleate (GMO) was calculated based on the experimental data of Katev *et al.*, which was generated by using the same *in vitro* digestion model [1]. The solubilization capacity of glycerol monolinoleate (GML) was estimated by multiplying the value for GMO with the coefficient obtained from the ratio of the solubilization capacities

LA/OA. The solubilization capacity of diolein was assumed to be the same as for dioleyl phosphatidylcholine, which was calculated based on the experimental data of Katev *et al* [1]. The solubilization capacity of dilinolein was estimated by multiplying the solubilization capacity of diolein with the LA/OA ratio. As the oleic acid and linoleic acid (as well as their derivatives) coelute in the GC analysis, a ratio of 1:2 (oleic to linoleic) was used to calculate the relative amounts of these components, which were then used to estimate the solubilization capacity. The 1:2 ratio of oleic to linoleic acid corresponds to the fatty acid profile of SFO. The solubilization capacities of the individual components are listed in Table S2.

Table S2. Solubilization capacities of the SFO lipolysis products, used for calculation of the solubilization capacities of the SFO digests.

Lipolysis product	Solubilization capacity, µg/mL fenofibrate per mM lipolysis product
Oleic acid	5.3
Linoleic acid	6.3
Glycerol monooleate	7.5
Glycerol monolinolein	8.9
Diolein	12.7
Dilinolein	15.0

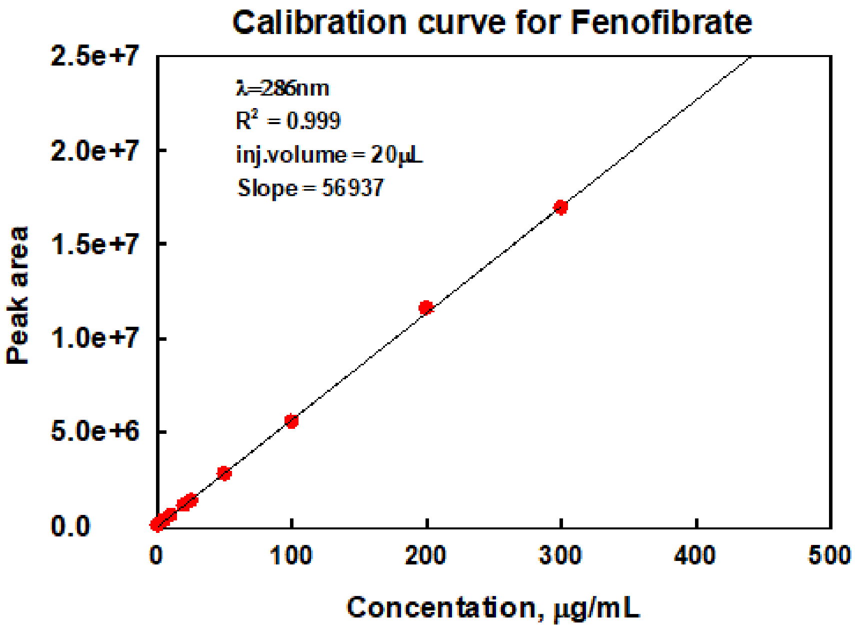

Figure S1. used for quantification of fenofibrate by HPLC-UV. Limit of quantification = 0.5 µg/mL.

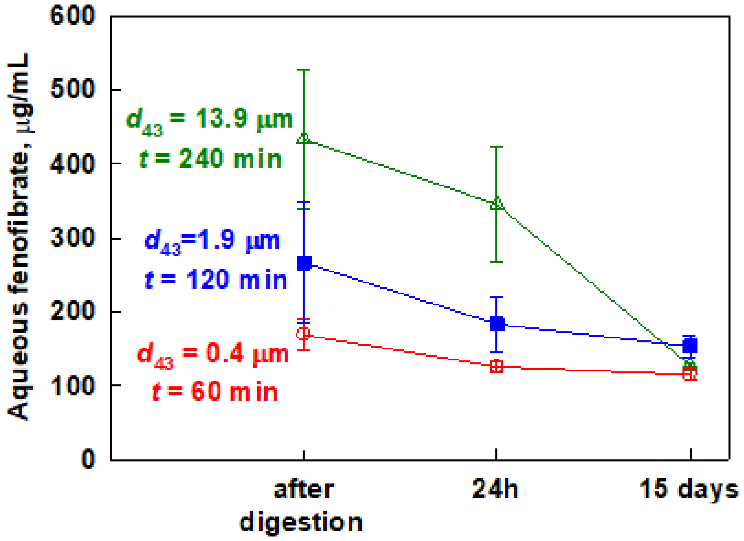

Figure S2. Aqueous fenofibrate after digestion of MCT emulsions with d_{43} = 0.4 µm (red circles), d_{43} = 1.9 µm (blue squares) and d_{43} = 13.9 µm (green triangles), measured immediately, 24 h, or 15 days after the experiment.

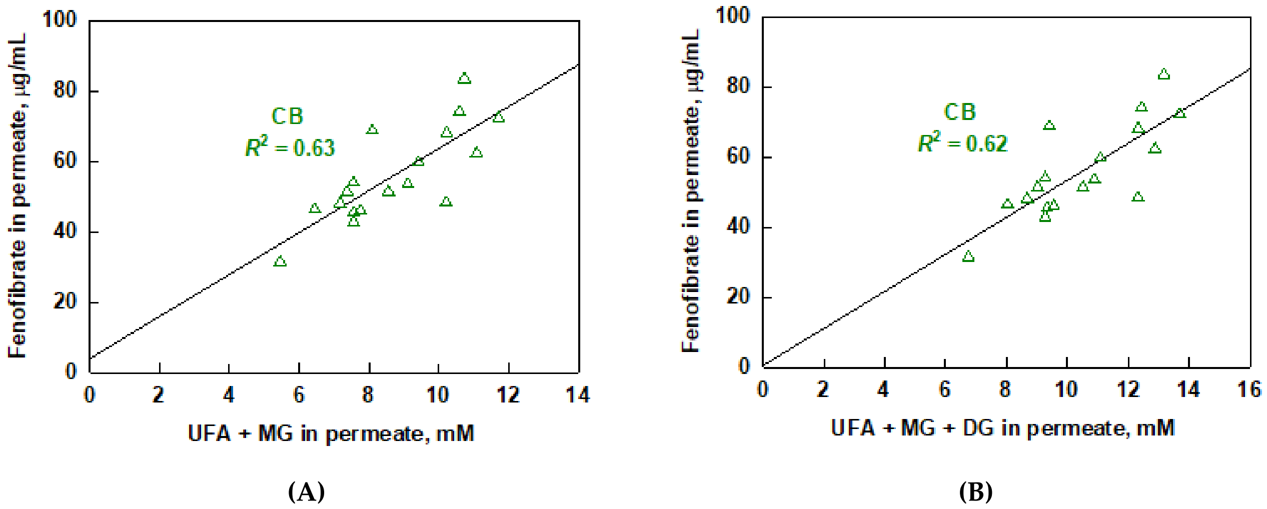

Figure S3. Aqueous fenofibrate as a function of (**A**) the unsaturated monoglycerides and unsaturated fatty acids in the permeate or (**B**) the unsaturated monoglycerides, unsaturated fatty acids and the diglycerides in the permeate, measured after digestion of CB emulsions with d_{43} = 2.0 µm.

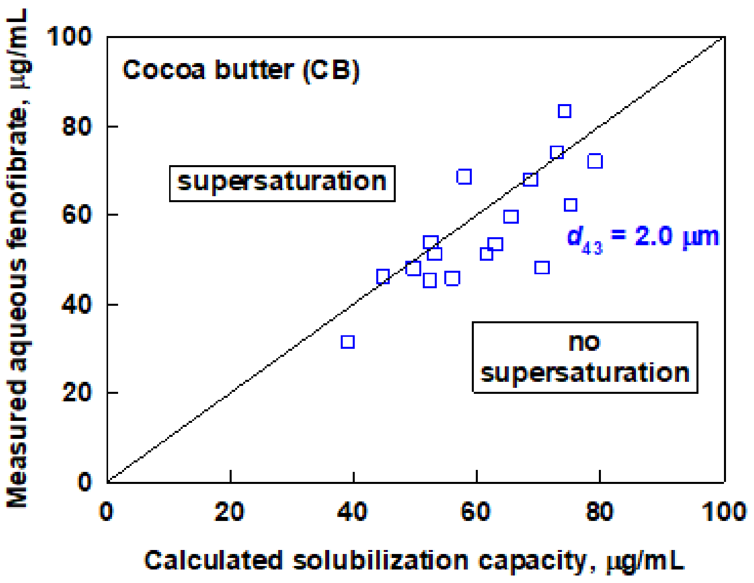

Figure S4. Aqueous fenofibrate as a function of the calculated solubilization capacity after digestion of CB emulsions with d_{43} = 2.0 µm (blue squares).

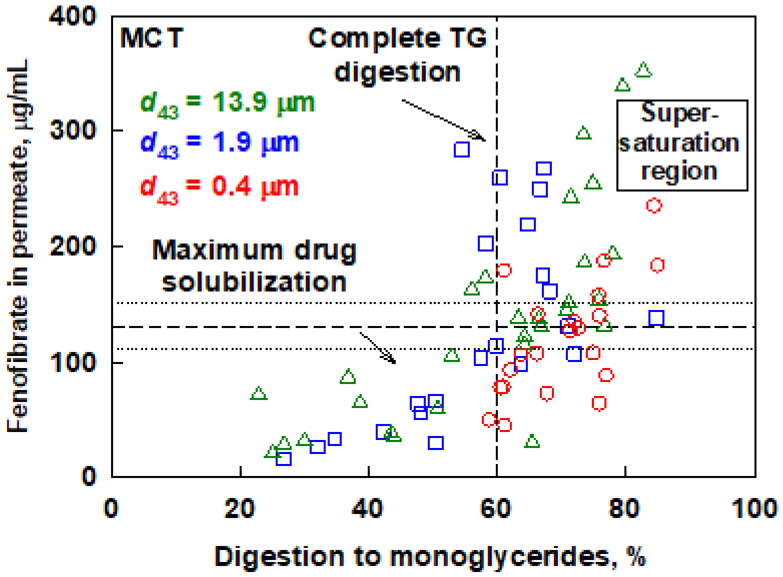

Figure S5. Aqueous fenofibrate as a function of the digestion to monoglycerides measured after *in vitro* digestion of MCT emulsions with d_{43} = 0.4 µm (red circles), d_{43} = 1.9 µm (blue squares) and d_{43} = 13.9 µm (green triangles). The line at 60 % digestion to MG corresponds to complete digestion of the oily TG phase.

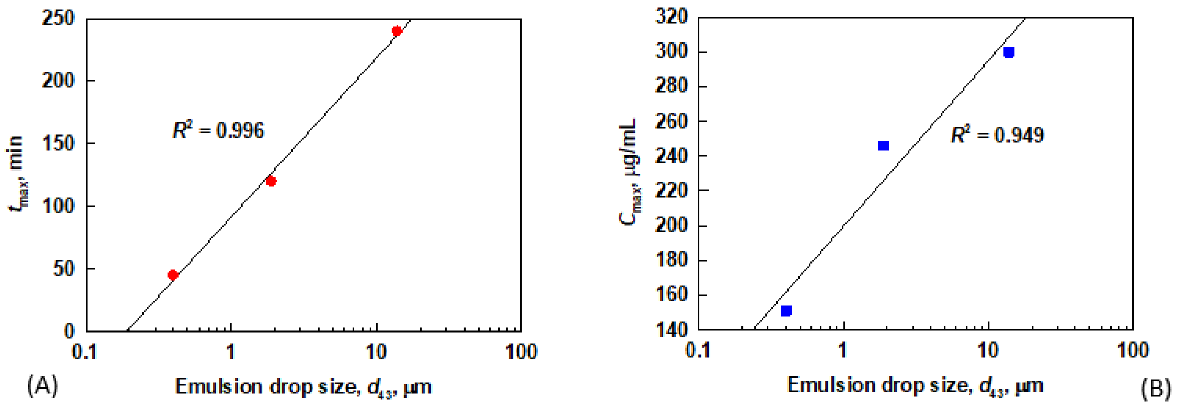

Figure S6. (**A**) Digestion time required to reach maximum aqueous fenofibrate concentrations (t_{max}) as a function of MCT emulsion droplet size, (**B**) maximum aqueous fenofibrate concentrations (C_{max}) as a function of MCT emulsion droplet size.

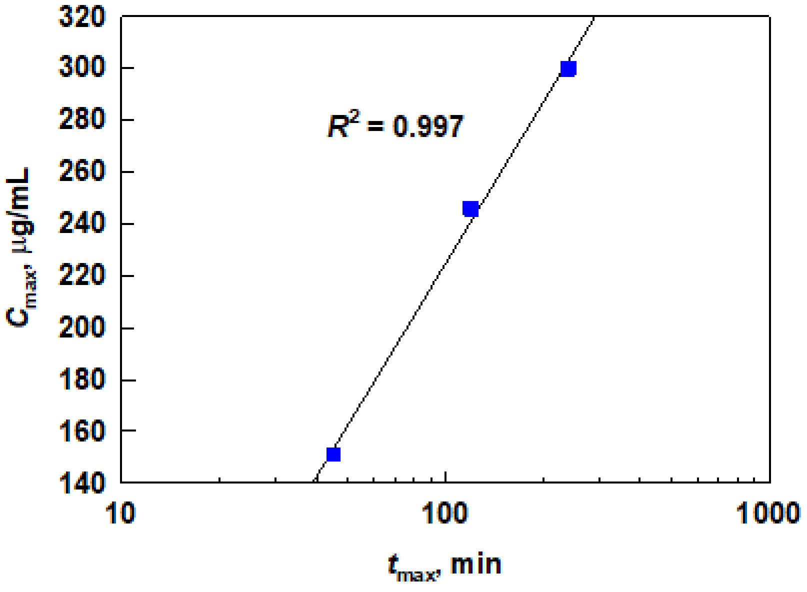

Figure S7. Supersaturation parameters after digestions of MCT emulsions: maximum fenofibrate concentration (C_{max}) as a function of the time required to reach that concentration (t_{max}).

References

1. Katev, V., Z. Vinarov, and S. Tcholakova, Mechanisms of drug solubilization by polar lipids in biorelevant media. *Eur J Pharm Sci*, **2021**. *159*: p. 105733.
